# Molecular Insights into the Role of the MET30 Protein and Its WD40 Domain in *Colletotrichum gloeosporioides* Growth and Virulence

**DOI:** 10.3390/jof11020084

**Published:** 2025-01-21

**Authors:** Fei Wu, Qianlong Sun, Longhui Huang, Sizhen Liu, Yue Chen, Xin Zhang, Chenggang Li, Sheng Guo, Xinqiu Tan

**Affiliations:** 1 LongPing Branch, College of Biology, Hunan University, Changsha 410125, China; wf1041225499@163.com (F.W.); huanlonhui@163.com (L.H.); l18942563793@163.com (S.L.); hnsfcy@126.com (Y.C.); 13699547753@163.com (S.G.); 2 Institute of Plant Protection, Hunan Academy of Agricultural Sciences, Changsha 410125, China; qianlongsun153@163.com (Q.S.); ailuo36@163.com (X.Z.); lcgag777@163.com (C.L.); 3 Yuelushan Laboratory, Changsha 410128, China

**Keywords:** *Phytopathogenic fungi*, MET30 protein, WD40 domain, pathogenicity, stress response

## Abstract

*Colletotrichum gloeosporioides* is a major phytopathogen responsible for anthracnose in *Capsicum annuum* (pepper) which leads to significant yield losses. At present, the molecular mechanism of *C. gloeosporioides* pathogenesis is not very clear. In this study, we focused on the MET30 protein and its key WD40 domain, with an emphasis on its role in the biological functions of *C. gloeosporioides*. Bioinformatics analysis revealed that the MET30 protein contains a conserved F-box domain and multiple WD40 repeats, which interact with other proteins to participate in various cellular processes, including nutrient acquisition, stress responses, and pathogenicity. Gene knockout and complementation experiments demonstrated that deleting the MET30 protein or its WD40 domain significantly reduced the rates of spore production and hyphal growth while increasing tolerance to environmental stresses such as high salinity and oxidative stress. Furthermore, pathogenicity assays revealed that the WD40 domain of the MET30 protein is crucial for regulating fungal pathogenicity, as mutants lacking WD40 domains presented increased virulence on pepper leaves. These findings suggest that the WD40 domain, in synergy with the MET30 protein, regulates the pathogenicity and stress response of *C. gloeosporioides*, provides new insights into the molecular mechanisms of anthracnose, and offers potential strategies for effective disease control.

## 1. Introduction

*Capsicum annuum* L. (pepper) is one of the most important economic crop species worldwide [1]. However, its production is significantly impacted by various plant pathogens that cause severe diseases. Among them, anthracnose caused by *Colletotrichum species* (Colletotrichum spp.) is widespread and economically devastating, with *Colletotrichum gloeosporioides* being the most prevalent species in tropical, subtropical, and temperate regions [2,3]. This pathogen infects a wide range of plants and causes significant losses in pepper production each year worldwide. To date, breeding peppers for disease resistance has relied mainly on race-specific resistance genes, which are easily overcome by emerging pathogenic strains. Thus, developing pepper cultivars with durable resistance is considered the most sustainable strategy for ensuring long-term pepper production [4]. However, the lack of extensive research on the pathogenicity genes and mechanisms of pepper anthracnose, combined with the limited genetic resources for resistance, has made finding pepper varieties resistant to anthracnose on a global scale challenging [5].

*C. gloeosporioides* is a hemibiotrophic pathogen with an infection cycle similar to that of *Magnaporthe oryzae*. During infection, conidia attach to the host plant surface and extend germ tubes, which then form an appressorium at the tip [6]. This structure penetrates the host cuticle and infiltrates epidermal cells, forming an infection vesicle within the host–cell wall membrane space [7], and subsequently differentiates into primary and secondary hyphae [6,8,9]. Studies have shown that fungal pathogenicity is associated with several phenotypes, such as spore size, hyphal growth rate, and cell wall integrity [10].

The pathogenic mechanism of *C. gloeosporioides* has become a research hotspot. CgOPT1 is involved in conidiation regulation in *C. gloeosporioides*; *cgopt1*-silenced mutants show delayed and reduced pathogenicity, and plants infected by this mutant produce high levels of indole-3-acetic acid (IAA) [11]. The membrane sensor protein CgSho1 works together with the adhesin CgMsb2 to regulate surface signal recognition in *C. gloeosporioides*. The absence of CgMsb2 leads to significant defects in appressorium formation, appressorium penetration, membrane permeability, and pathogenicity, whereas the loss of CgSho1 causes defects in hyphal expansion after infection [12]. The MAPK response gene *Cgl-SLT2* regulates spore formation, polar growth, appressorium formation, and pathogenicity, and a loss of *Cgl-SLT2* leads to defects in vegetative growth, appressorium formation, and pathogenicity in *C. gloeosporioides* [13]. *CgRac1*, a gene involved in regulating morphology, nuclear division, and appressorium formation in *C. gloeosporioides*, displays a double germ tube phenotype in the *CA-CgRac1* mutant strain, in which establishing and maintaining cell polarity is difficult and conidiation and hyphal attachment are reduced; thus, normal appressoria cannot form, resulting in reduced pathogenicity [14]. Conidial morphology 1 (COM1), a protein involved in conidial morphology, affects conidial germination, germ tube development, and appressorium formation, thus influencing virulence [15]. Nitrogen metabolism genes such as *GDH2*, *GS1*, *GLT*, and *MEP* are differentially expressed during *C. gloeosporioides* colonisation and play roles in its pathogenicity. The deletion of these genes weakens the pathogenicity of *C. gloeosporioides* [16]. Cg2LysM is involved in conidiation, appressorium formation, and infection spread, as well as in regulating the synthesis of melanin in *C. gloeosporioides*. Cg2LysM can bind chitin, regulates reactive oxygen species (ROS) production, and affects the expression of host defence-related genes such as *HbPR1*, *HbPR5*, *HbNPR1*, and *HbPAD4* during infection [17]. CrzA stimulates the activities of chitin synthase and glucan synthase, whereas SltA regulates the expression of the MAP kinase pmk1 and the acetyltransferase cat1, wherein all of these factors impact *C. gloeosporioides* colonisation [18]. CgareA, a global nitrogen regulatory factor, significantly reduces vegetative growth, slightly decreases the conidial germination rate, and misregulates nitrogen metabolism-related genes in the mutant strain, leading to weakened pathogenicity [19].

F-box/WD40 (FBXW) proteins, a subclass of the F-box protein family, contain a C-terminal WD40 domain [20]. In model fungi such as *Saccharomyces cerevisiae* and *Candida albicans*, the FBXW protein CDC4 is involved in processes such as G1‒S phase transition, morphogenesis, temperature stress tolerance, amino acid biosynthesis, and calcium sensitivity [21]. Additionally, CDC4 is essential for virulence and sexual reproduction in the pathogenic fungus *Cryptococcus neoformans* [22]. In *M. oryzae*, the F-box proteins MoFbx15 and MoCdc4 are crucial for full pathogenicity, spore differentiation, germination, and appressorium formation [23]. However, the specific contributions of the WD40 domain to these processes remain unknown. The WD40 domain in FBXW family members provides a large surface area for protein‒protein interactions (PPIs) [24], suggesting additional roles beyond ubiquitination. Previous studies have shown that WD40 proteins, such as CPCb in *Aspergillus fumigatus* [25] and FSPR1 in *Fusarium oxysporum* [26] and *Fusarium graminearum* [27], are associated with fungal virulence. However, the role of FBXW proteins in *C. gloeosporioides* remains unknown, and their functional significance has yet to be determined.

In this study, we focused on the FBXW protein MET30 in *C. gloeosporioides* and its WD40 domain, aiming to explore its roles in fungal growth, stress tolerance, and pathogenicity. By analysing the biological functions of the MET30 protein and its WD40 domain, we sought to reveal their critical role in the development of fungal virulence and the underlying regulatory mechanisms. This research will not only deepen our understanding of the role of the WD40 domain in fungal biology but will also provide novel molecular targets and strategies for controlling pepper anthracnose.

## 2. Materials and Methods

### 2.1. Strain and Culture Conditions

The wild-type (WT) strain *C. gloeosporioides* CSLL-11 used in this study was isolated from a sample in Changsha, Hunan Province. All strains were cultured on potato dextrose agar (PDA) plates (Solarbio, P8931, Beijing China) at 28 °C. Liquid complete medium (CM) was used to obtain conidia and mycelia, with the latter used for DNA and RNA extraction (Table S1).

### 2.2. Bioinformatics Analysis

On the basis of the CgMET30 sequencing results, online tools such as SMART (https://smart.embl.de/) were used to predict its protein domains. Additionally, the three-dimensional structure of CgMET30 was predicted using SWISS-MODEL (https://swissmodel.expasy.org/). The coding sequence can be found in the table (Table S2).

### 2.3. Mutant Generation and Complementation

A *CgMET30* gene knockout vector was constructed via homologous recombination to delete *CgMET30* in a targeted manner (Figure S1). Protoplast transformation was performed to introduce the vector into the WT strain. Positive transformants were identified and verified using Southern blot analysis, and the deletion mutant was named Δ*CgMet30*.

To create complementation strains, constructs containing the full-length *CgMET30* gene and a version of the gene lacking the WD40 domain were introduced into the Δ*CgMet30* strain via protoplast transformation. The positive transformants were verified via PCR, and the complementation mutants were named Δ*CgMet30*/CgMET30 and Δ*CgMet30*/CgMET30Δ*WD40*, respectively.

### 2.4. Southern Blotting

Following Cariani’s method, 15 μg of DNA was digested using restriction endonucleases *EcoR* V and *Xba* I, blotted onto nylon for nucleic acid transfer (GE Healthcare Amersham Hybond-N™, Pittsburgh, PA, USA), and hybridised with DIG-labelled probes using the Dig High Prime DNA Labeling and Detection Starter Kit I (Roche, Penzberg, Germany), according to the manufacturer’s instructions [28].

### 2.5. Assessment of the Growth Rate, Sporulation, and Conidial Germination

To evaluate the role of CgMET30 in vegetative growth, identical mycelial plugs were inoculated onto PDA, OM, SDC, V8, and CM agar plates and incubated at 28 °C for 5 days (Table S1). The colony diameters were measured and statistically analysed. All growth assays were repeated three times, with three replicates on each occasion [29].

To assess sporulation, identical mycelial plugs from each strain were cultured in 100 mL of CM at 28 °C for 3 days with shaking at 200 rpm. The conidia were collected by filtering the culture through sterile filter paper, and after centrifugation at 8000 rpm, the conidia were washed three times with sterile water and used to prepare a conidial suspension. The spore concentration was determined using a haemocytometer. All the experiments were repeated three times with three replicates on each occasion.

A conidial suspension (1 × 10^5^ cfu/mL) was prepared and added to hydrophobic coverslips dropwise at 28 °C in the dark. After 12 h, conidial germination and appressorium formation were observed and statistically analysed.

### 2.6. Evaluation of Stress Tolerance

Single-colony mycelial plugs (5 mm in diameter) were excised using a punch, inoculated on PDA media containing 0.7 M NaCl, 0.6 M KCl, 1 M sorbitol, SDS, or 2,4,6 mM hydrogen peroxide, and incubated at 28 °C for 5 days, after which the rate of growth inhibition for each treatment was calculated to determine the effects of different stress conditions on colony growth [30].

### 2.7. Protoplast Release Assay

Single-colony mycelial plugs (5 mm in diameter) were excised using a punch, with ten plugs taken from each strain. Each plug was cut into four equal pieces, inoculated into 5× YEG liquid medium, and cultured at 28 °C and 120 rpm for 12 h. After cultivation, mycelia were collected for protoplast preparation. The mycelia were treated with 1% lysing enzyme at 30 °C and 60 rpm for 2 h. The protoplast suspension was filtered through a three-layer Miracloth membrane, and then the number of protoplasts was determined using a haemocytometer and photographs were taken.

### 2.8. Pathogenicity Tests

The pathogenicity of the strains was evaluated on the same compound leaf of pepper plants at the 5–6 leaf stage.

Mycelial plugs (5 mm in diameter) from 3-day-old colonies were inoculated onto detached pepper leaves and incubated at 25 °C in the dark with 80% humidity. After 3 days, photographs were taken, and the lesion sizes were measured.

A conidial suspension (1 × 10^5^/mL) was prepared and symmetrically inoculated onto detached pepper leaves (20 µL per side). After incubation at 25 °C with 80% humidity in the dark for 5 days, lesion sizes were measured and recorded.

### 2.9. Trypan Blue Staining

Compound leaves from pepper plants at the 5–6-leaf stage were inoculated at the same position with a spore suspension (1 × 10^5^ cfu/mL). Each leaf was symmetrically inoculated at the same location with 20 µL of the spore suspension, after which the tray was sealed, and the samples were incubated at 25 °C for 5 days. After 12 h of staining with lactophenol blue solution (each 100 mL of solution contained 10 mL of phenol, 10 mL of glycerol, lactic acid, and 2–5 g of Trypan blue), the leaves were decolourised with water and trichloroacetic acid decolourisation solution (250 g of chloral hydrate dissolved in 100–150 mL of sterile water) and photographed.

### 2.10. Quantitative Reverse Transcription Polymerase Chain Reaction (RT‒qPCR)

Total RNA was extracted using the Total RNA Kit II (Omega, Norwalk, CT, USA). Reverse transcription was performed using the MonScript™ RTIII Super Mix (Monad, Wuhan, China), and qPCR was conducted with ChamQ Universal SYBR qPCR Master Mix (Vazyme, Nanjing, China). The *C. gloeosporioides* Actin gene was used as the internal control. The expression of genes related to cell wall integrity and conidiation was detected using a CFX96 instrument (Bio-Rad, Hercules, CA, USA). Relative transcription levels were calculated using the 2^−ΔΔCt^ method (Tables S3–S5).

### 2.11. Statistical Analysis

All the experiments were performed with three biological replicates. The data are presented as the means ± standard errors (means ± SEMs). Statistical analyses were performed using GraphPad 8.0 software, and independent sample t-tests were used to determine significance, with *p* < 0.05 considered to indicate a significant difference.

## 3. Results

### 3.1. Characterisation of CgMET30 in C. gloeosporioides

**Figure 1:**
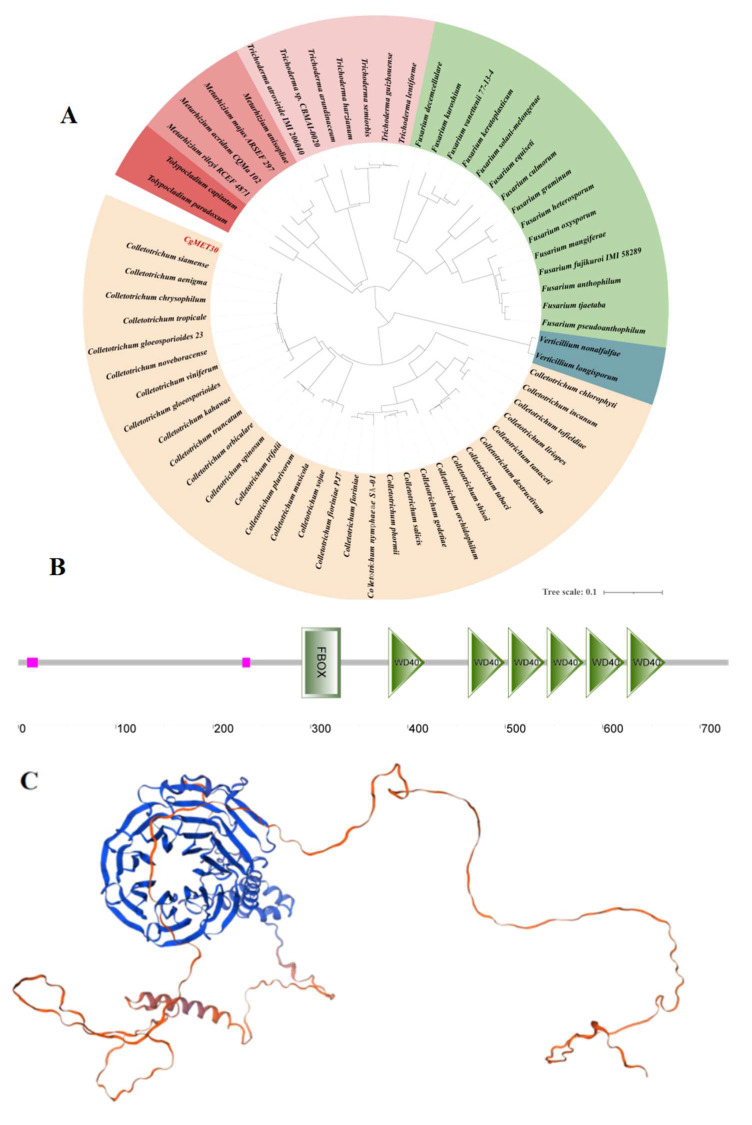
The CgMET30 protein structure was predicted and analysed. (**A**) The predicted structural domains of the CgMET30 protein. (**B**) The predicted tertiary structure of the CgMET30 protein. (**C**) The predicted tertiary structure of the CgMET30 protein.

Through bioinformatics analysis, the CgMET30 protein is highly conserved within the Ascomycota phylum, with an over 90% homology to pathogenic fungi such as Aspergillus fumigatus, Fusarium, Neurospora crassa, and Verticillium dahlia (Figure 1A). CgMET30 was identified as a typical F-box/WD40 domain protein belonging to the FBXW family. This protein contains an F-box domain and six WD40 repeat sequences (Figure 1B). SMART analysis predicted that the WD40 domain in CgMET30 adopts the characteristic β-propeller structure (Figure 1C).

### 3.2. Generation of Gene Knockout and Complementation Strains

To investigate the function of the *CgMet30* gene and its WD40 domain in *C. gloeosporioides*, a *CgMet30* knockout vector was constructed, and four single-copy knockout strains were obtained via PEG-mediated protoplast transformation (Figure S2). Additionally, to explore the critical role of the WD40 domain in the functionality of the MET30 protein, two complementation strains were generated: one with the full-length *CgMet30* gene and the other with a gene with WD40 domain deletion (Figure S3).

### 3.3. Role of the WD40 Domain in the CgMET30 Protein in the Regulation of Vegetative Growth and Conidial Formation

**Figure 2:**
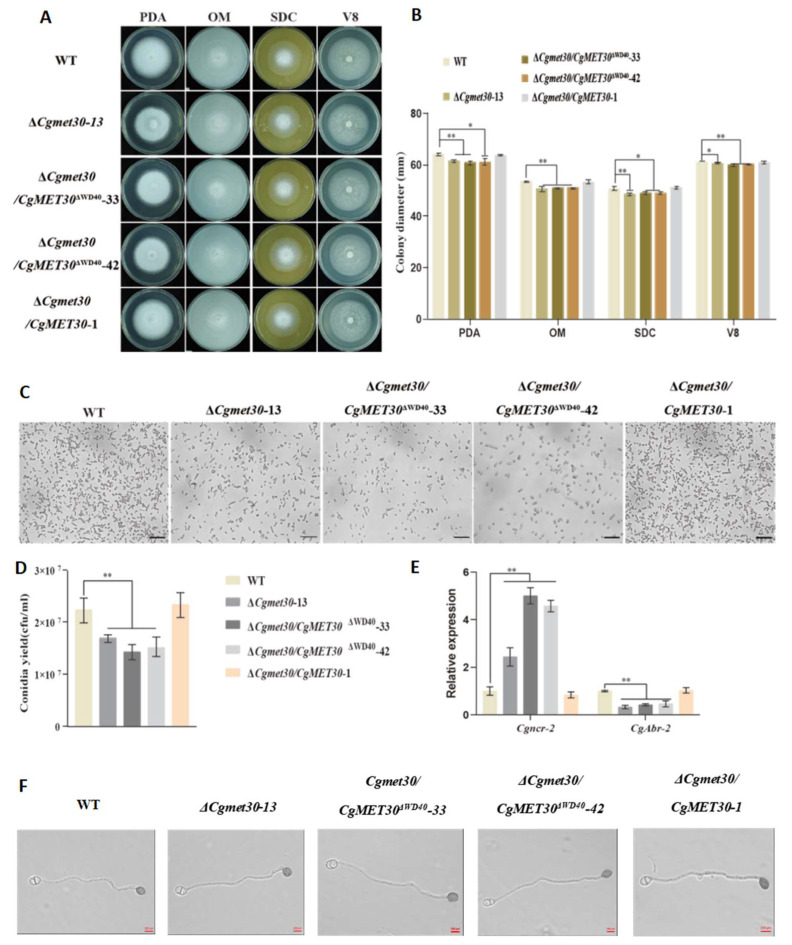
CgMET30 is involved in nutritional growth. (**A**,**B**) Growth rates on PDA, OM, SDC, and V8 media. (**C**,**D**) Comparison of conidia yields. (**E**) Expression levels of selected conidia-related genes detected via RT–qPCR. (**F**) Morphological observations of the formed appressoria. Observe using a 40x microscope. Scale bars represent 200 μm. Error bars represent ± SD of three replicates, asterisks (*) indicate significant difference (*t*-test *p* < 0.05), asterisks (**) indicate extremely significant difference (*t*-test, *p* < 0.01).

The growth analysis revealed that the deletion of the *CgMET30* gene significantly inhibited the growth of *C. gloeosporioides* colonies on various media (PDA, V8, SDC, and OM) which decreased to 90.51 ± 3.23% of the WT; the deletion of the WD40 domain resulted in the growth of *C. gloeosporioides* on these media reaching only 89.72% ± 2.92% of the WT growth. These findings indicate that the WD40 domain plays a crucial role in the normal vegetative growth of *C. gloeosporioides* (*p* < 0.05, Figure 2A,B).

Conidial production by both the Δ*CgMet30* and Δ*WD40* mutants was significantly reduced, with the WD40 domain deletion mutant showing a more pronounced effect. Specifically, under identical conditions, conidial production by the Δ*CgMet30* mutant was 24.19% lower than that by the WT strain, whereas the Δ*WD40* mutant exhibited a reduction of 33.92 ± 1.97% (Figure 2C,D). Although conidial production by the Δ*CgMet30* and Δ*WD40* mutants was significantly reduced, conidial germination rate, formation time, and the rate of appressoria were not affected (Figure 2F).

RT‒qPCR analysis was performed on relevant genes to further investigate the molecular mechanisms by which the CgMET30 protein and its WD40 domain regulate conidial formation. The gene Cgncr-2, which encodes a serine/threonine kinase required to inhibit conidial formation, was significantly upregulated in the Δ*CgMet30* and Δ*WD40* mutants, with expression levels 2.43 times and 4.78 ± 0.22 times greater than those in the WT, respectively. Conversely, CgAbr-2, a gene that is active during conidiation, was significantly downregulated in both the Δ*CgMet30* and Δ*WD40* mutants, with expression levels reduced to 0.33 times and 0.44 ± 0.02 times that in the WT, respectively, indicating that deletion of CgMET30 and the WD40 domain affects conidial production (Figure 2E).

In summary, the WD40 domain is an important part of the MET30 protein and plays critical roles in regulating vegetative growth and conidial production in *C. gloeosporioides* but does not affect the formation of appressoria. Therefore, it directly impacts the fungal development and the ability to form conidia.

### 3.4. Core Role of the C. gloeosporioides WD40 Domain in the CgMET30-Mediated Stress Response

**Figure 3:**
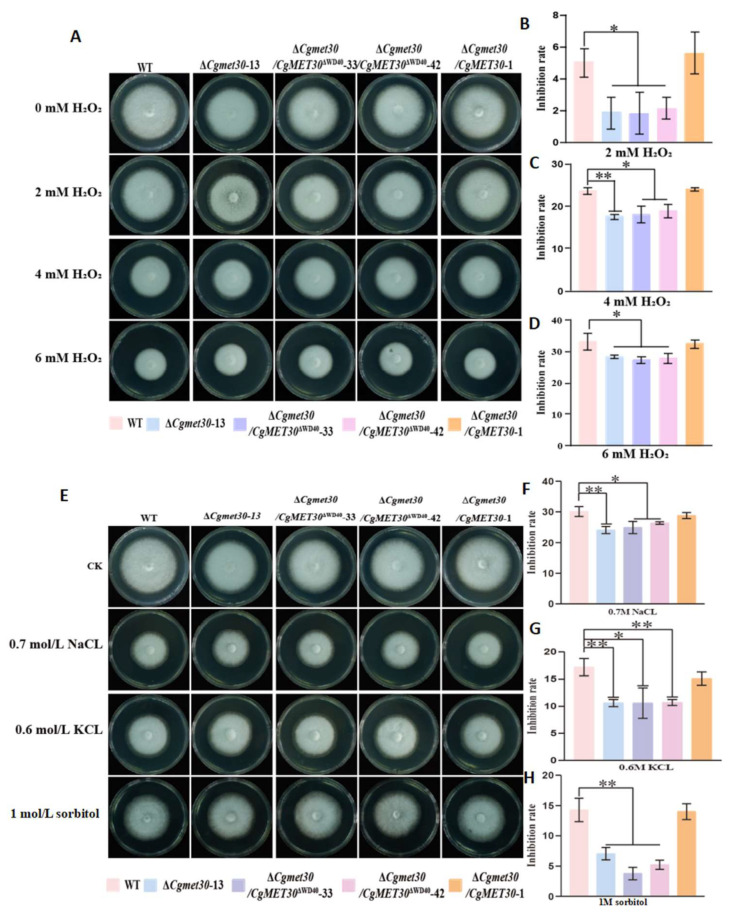
Stress tolerance test. (**A**–**D**) Stress tolerance of all the strains to ions and salt. (**E**–**H**) Stress tolerance of all the strains to oxidative stress. Similar results were obtained after three repetitions of the procedure. Error bars represent ± SD of three replicates and asterisks (*) indicate significant difference (*t*-test *p* < 0.05), asterisks (**) indicate extremely significant difference (*t*-test *p* < 0.01).

Stress tolerance experiments showed significantly enhanced growth in ΔWD40 mutants under NaCl (0.7 M), KCl (0.6 M), sorbitol (1 M), and H_2_O_2_ (2,4,6 mM) conditions compared to WT. These results suggest that the WD40 domain plays a crucial regulatory role in the CgMET30 protein by inhibiting specific stress response pathways, thereby affecting the tolerance of *C. gloeosporioides* to environmental stresses (*p* < 0.05, Figure 3).

These findings highlight the importance of the WD40 domain in regulating the adaptive responses of fungi to stress, potentially by modulating pathways that suppress excessive growth under adverse conditions.

### 3.5. The Regulatory Role of the C. gloeosporioides CgMET30 WD40 Domain in the Maintenance of Cell Wall Integrity

**Figure 4:**
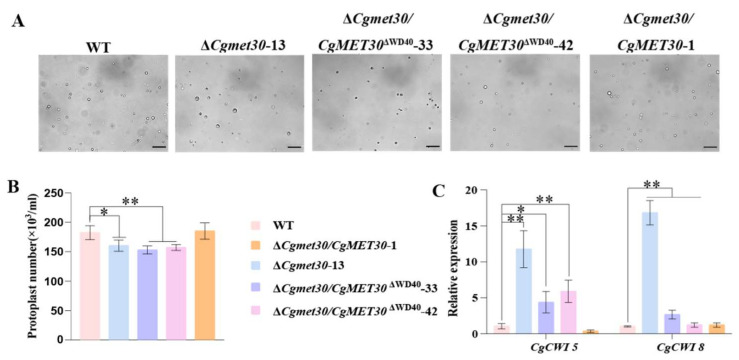
CgMET30 is involved in maintaining cell wall integrity. (**A**,**B**) Number of protoplasts released after mycelia were treated with equal amounts of lysing enzymes (scale bar, 200 μm). (**C**) RT–qPCR was used to measure the expression levels of cell wall synthase-related genes. Similar results were obtained after three repetitions of the procedure. Error bars represent ± SD of three replicates, asterisks (*) indicate significant difference (*t*-test *p* < 0.05),asterisks (**) indicate extremely significant difference (*t*-test, *p* < 0.01).

To investigate the role of CgMET30 and its WD40 domain in maintaining cell wall integrity in *C. gloeosporioides*, protoplast release assays were conducted by treating fungal mycelia with cell wall-degrading enzymes. Compared with the WT and complementation strains, the Δ*CgMet30* and Δ*WD40* mutant strains released significantly fewer protoplasts, indicating an increased resistance of their cell walls to enzymatic degradation. These findings suggest that the cell walls of these mutants were more challenging to break down (Figure 4A,B).

The cell wall synthesis-related genes *CgCWI5* and *CgCWI8* were significantly upregulated in the mutants. Specifically, *CgCWI5* expression in the Δ*CgMet30* mutant was 11.75 times greater than that in the WT, whereas in the Δ*WD40* mutant, *CgCWI5* expression was 5.13 ± 0.75 times greater. The expression level of *CgCWI8* in the Δ*CgMet30* mutant was 16.81 times greater than that in the WT, and in the Δ*WD40* mutant, the expression level of *CgCWI8* was 2.33 ± 0.33 times greater (Figure 4C).

Our findings strongly indicate that CgMET30 and its WD40 domain are pivotal in maintaining *C. gloeosporioides* cell wall integrity. This is likely achieved by regulating genes involved in cell wall synthesis and restructuring, an important insight that informs our understanding of the cellular processes involved.

### 3.6. Key Regulatory Role of the CgMET30 WD40 Domain -C. gloeosporioides Pathogenicity

**Figure 5:**
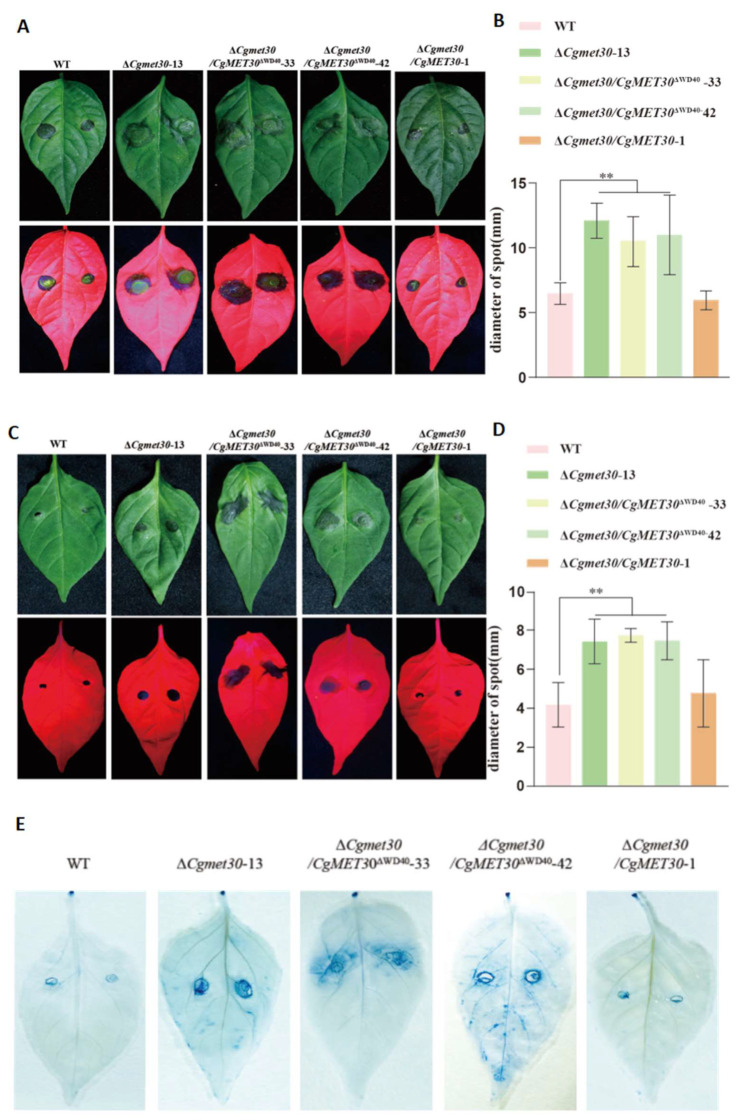
Pathogenicity assay. (**A**,**B**) Inoculation of mycelia to assess pathogenicity. (**C**,**D**) Inoculation of conidia to assess pathogenicity. (**E**) Mycelium expansion was observed via Trypan blue staining. Similar results were obtained after three repetitions of the procedure. Error bars represent ± SD of three replicates and asterisks (**) indicate extremely significant difference. (*t*-test, *p* < 0.05).

The pathogenicity assays revealed that mutants lacking the full-length CgMET30 protein or the protein lacking the WD40 domain caused significantly larger lesions on pepper leaves than did the WT strain, indicating that these mutants exhibit increased virulence. Specifically, the lesion sizes caused by the Δ*CgMet30* and Δ*WD40* mutants were 1.87 and 1.70 times larger than those caused by the WT strain, respectively, indicating greater aggressiveness and faster spread (Figure 5A–D). Trypan blue staining confirmed this observation, showing that the mutants spread rapidly within the host tissue and exhibited increased invasiveness (Figure 5E).

These results suggest that the WD40 domain in CgMET30 may limit pathogenicity by suppressing specific virulence pathways. In combination with the results of the stress tolerance experiments, the enhanced pathogenicity of the mutants is likely due to their increased adaptability and invasiveness, particularly in response to environmental stress, which allows them to better withstand host defences and spread more effectively.

## 4. Discussion

WD40 repeat (WDR) proteins, characterised by the presence of WD40 sequences, were first identified in the Gβ subunit of heterotrimeric G proteins and the CDC4 protein in 1986. WDR proteins are named after their conserved tryptophan (W)-aspartic acid (D) dipeptide moiety and a sequence of approximately 40 amino acid residues [31]. In 1999, Smith et al. [32] further refined the definition of this motif, where the N-terminal 11–24 residues contain glycine (G)-histidine (H) repeats and the C-terminal end contains the W-D dipeptide. These sequences typically consist of 44–60 amino acid repeats. WD40 proteins are found in a variety of eukaryotic cells, with a greater abundance in fungi than in animals and plants. Structurally, WD40 proteins form β-propeller architectures composed of 4–8 blades, providing a platform for protein‒protein or protein‒DNA interactions [33,34]. WD40 proteins are involved in various signalling pathways, including DNA damage recognition and repair [35], ubiquitin signalling [36], and immune-related pathways [26]. Given their unique structure, WD40 proteins are considered promising targets for PPI inhibitor drugs [27].

In this study, we found that deleting the WD40 domain significantly inhibited colony growth and conidial production in *C. gloeosporioides*. This inhibitory effect suggests that the WD40 domain plays essential roles in cell division and growth by stabilising interactions with related proteins. For example, in *M. oryzae*, the WD40 family protein MoCdc4 regulates appressorium formation, directly impacting the pathogen’s infective capacity [23]. Similarly, in *S. cerevisiae*, the WD40 family protein Cdc4 plays a key role in cell cycle regulation, and the loss of this domain results in arrested cell growth and abnormal cell division [37,38]. Additionally, in *F. oxysporum*, the WD40 family protein FSPR1 is critical for spore formation and hyphal growth, with deletion mutants displaying significant developmental defects [26]. Consistent with these findings, the WD40 domain deletion mutants in this study presented more pronounced growth inhibition and defects in spore formation, further validating the pivotal role of this domain in fungal development. These results indicate that the WD40 domain of the MET30 protein is crucial for cell growth and development and likely functions as a core component within the regulatory network that governs *C. gloeosporioides* growth.

In this study, the negative regulatory role of the WD40 domain in the MET30 protein-mediated stress response was also revealed and is one of the critical contributions of this research. The experimental results demonstrated that deleting the WD40 domain significantly increased *C. gloeosporioides* tolerance to various environmental stresses, particularly high salinity and oxidative stress. Similar phenomena have been observed in other fungi. For example, the WD40 family protein CpcB in *A. fumigatus* has been shown to play a critical role in regulating stress responses, and its deletion mutants exhibit increased tolerance to oxidative stress [25]. In contrast, the results of our study suggested that the MET30 protein in *C. gloeosporioides* may limit the expression of certain stress response genes via its WD40 domain, thereby reducing the stress tolerance. This negative regulatory mechanism in plant pathogenic fungi offers a new perspective for understanding the complexity of fungal stress responses and highlights the functional diversity of FBXW proteins across fungal species. Furthermore, the WD40 domain-containing protein Msi1 in *C. neoformans* has displayed similar negative regulatory characteristics [34], which affect the response of this fungus to osmotic and oxidative stress. These findings further support our conclusion regarding the critical role of the WD40 domain in regulating fungal stress responses.

The *C. gloeosporioides* infection of the host leads to the differentiation of primary and secondary hyphae within its epidermal cells. The primary hyphae, which are located within the epidermal cells, are short lived and involved primarily in nutrient acquisition. Secondary hyphae invade mesophyll cells and infect neighbouring cells, gradually spreading throughout the host tissue [7]. The pathogen then secretes cell wall-degrading enzymes or toxins, causing host tissue necrosis. The extension of germ tubes and hyphae, as well as the generation of turgor pressure, are closely related to cell surface expansion and cell wall integrity [39]. The protoplast release experiments in this study demonstrate that the cell wall of the WD40 domain mutant exhibits increased resistance to enzymatic degradation, and the expression levels of cell wall biosynthesis-related genes are significantly upregulated. This suggests that the WD40 domain plays an important role in maintaining cell wall integrity. This effect may be mediated through the regulation of a specific gene, providing new insights for further investigation into the function of the WD40 domain.

With respect to pathogenicity, the regulatory role of the WD40 domain in MET30 protein function is particularly prominent. The results of the pathogenicity experiments in this study revealed that the deletion of the WD40 domain significantly increased the virulence of *C. gloeosporioides* to pepper, with the mutants causing noticeably larger lesions on the pepper leaves than the WT strain did. These findings suggest that the WD40 domain may suppress virulence by inhibiting genes or signalling pathways related to pathogenicity, thereby limiting pathogen virulence. Similar regulatory mechanisms have been observed in other plant pathogenic fungi. For example, the WD40 family protein MoCdc4 in *M. oryzae* regulates appressorium development, directly affecting the infection ability of the pathogen [23]. Similarly, in *F. graminearum*, the WD40 domain protein Fsr1 has been found to regulate fungal virulence, with deletion mutants showing an enhanced ability to infect host plants [27]. Moreover, in *Colletotrichum graminicola*, the WD40 family protein RACK1 influences virulence by regulating the synthesis of secondary metabolites [40]. Consistent with these studies, the WD40 domain deletion mutants in our study presented increased virulence, confirming the critical role of this domain in regulating fungal virulence. Our results suggest that the WD40 domain of the MET30 protein not only plays key roles in regulating fungal growth and the stress response but also functions through a complex gene regulatory network to limit pathogenicity. This ensures that host plant defence mechanisms can partially function, which has significant implications for our understanding of plant‒pathogen interactions.

The structure‒function relationship of the WD40 domain in the MET30 protein is crucial for understanding its diverse biological roles. In this study, through a detailed analyses of the WD40 domain deletion mutants, the central importance of this domain in the function of MET30 was revealed. The deletion of the WD40 domain not only significantly inhibited fungal growth and conidial formation but also markedly enhanced the stress tolerance and pathogenicity of *C. gloeosporioides*. Compared with other fungal proteins containing WD40 domains, the WD40 domain of MET30 in *C. gloeosporioides* has more diverse functions. For example, in *S. cerevisiae*, the WD40 protein Cdc4 mainly regulates the cell cycle through interactions with the SCF complex [21], whereas the WD40 protein SWD1 in *Aspergillus flavus* is essential for the biosynthesis of numerous primary and secondary metabolites, contributing to reshaping metabolic processes and potentially suppressing fungal virulence by inducing hyphal apoptosis via sphingolipid pathways [36]. These studies further support the multifunctionality of WD40 domain proteins within fungal protein networks. In this study, the WD40 domain of the MET30 protein demonstrated broad regulatory effects in *C. gloeosporioides*, encompassing critical biological processes such as growth and pathogenicity. This discovery opens new directions for future research into the role and function of the WD40 domain in fungal biology, highlighting its potential as a regulatory hub within fungal protein interaction networks.

In conclusion, this study systematically revealed the multiple biological functions of the MET30 protein and its WD40 domain in *C. gloeosporioides*, particularly its vital regulatory roles in vegetative growth, stress tolerance, and pathogenicity. The WD40 domain performs diverse and essential regulatory functions in these processes, serving as a critical module for the various biological effects of MET30. These findings expand our understanding of the roles of FBXW proteins in plant pathogenic fungi and provide new insights into their functional mechanisms in other pathogens. Future research could explore the specific role of the WD40 domain in protein degradation pathways, particularly its involvement in the ubiquitin‒proteasome system, to further clarify its molecular mechanisms in pathogen‒host interactions. Additionally, these fundamental discoveries may inform the development of anthracnose control strategies, such as designing antifungal drugs that target the PPIs mediated by the WD40 domain or enhancing plant disease resistance. This research provides a solid theoretical foundation for understanding the pathogenic mechanisms of *C. gloeosporioides* and offers potential solutions for disease control in agricultural applications.

## 5. Conclusions

In this study, the multiple biological functions of the MET30 protein and its WD40 domain in *C. gloeosporioides* were thoroughly explored, and the critical roles of the WD40 domain in regulating fungal growth, stress response, and pathogenicity were revealed. Our research demonstrated that the WD40 domain plays essential roles in cell growth and conidial formation, negatively regulates the stress response, and limits virulence, thereby significantly influencing the biological behaviour of this pathogen. These findings expand our understanding of FBXW proteins in plant pathogenic fungi and provide a new theoretical foundation for future studies.

## Data Availability

The original contributions presented in this study are included in the article/Supplementary Materials. Further inquiries can be directed to the corresponding author.

## Supplementary Materials

The following supporting information can be downloaded at https://www.mdpi.com/article/10.3390/jof11020084/s1: Figure S1. Diagram of homologous recombination structure. Figure S2. Gene knockout and validation. (**A**) The upstream arm of the knockout plasmid was identified by double enzyme digestion, where M is a 5000 bp marker, 1 is the plasmid pCX62::*CgMET30*-Up digested by Xho I and Hind III, and 2 is the pCX62::*CgMET30*-Up plasmid. (**B**) The downstream arm of the knockout plasmid was identified by double enzyme digestion, where M is a 5000 bp marker, 1 is the pCX62::*CgMET30*-Down plasmid, and 2 is the plasmid pCX62::*CgMET30*-Down digested by Spe I and Not I. (**C**) Transformant screening via PCR using the *CgMET30*-F1/*CgMET30*-R primer pairs, where M is a 5000 bp marker, 1~23 are *CgMET30* partial knockout transformants, 24 is the pCX62::*CgMET30* plasmid, and 25 is WT CSLL-11 genomic DNA. (**D**) Transformant screening via PCR using the *HPH*-F/*HPH*-R primer pairs, where M is a 2000 bp marker, 1~23 are *CgMET30* partial knockout transformants, 24 is the pCX62::CgMET30 plasmid, and 25 is WT CSLL-11 genomic DNA. (**E**) Hph probe hybridisation, where 1~5 are transformants Δ*Cgmet30*-2, Δ*Cgmet30*-10, Δ*Cgmet30*-13, Δ*Cgmet30*-17, and Δ*Cgmet30*-19, respectively, 6 is the WT, and 7 is the knockout vector. (**F**) *CgMET30* probe hybridisation, where 1~4 are transformants Δ*Cgmet30*-2, Δ*Cgmet30*-13, Δ*Cgmet30*-17, and Δ*Cgmet30*-19, respectively, 4 is the WT, and 5 is the *CgMET30* PCR product. Figure S3. (**A**) Complementary *CgMET30* transformants were screened via PCR with the primers *CgMET30*-1F and *CgMET30*-R. (**B**) Complementary *CgMET30* transformants were screened via PCR with the primers *G418*-F and *G418*-R primers. (**C**) Complementary *CgMET30*Δ*WD40* transformants were screened via PCR with the primers *CgMET30*-1F and *CgMET30*-R. (**D**) Complementary *CgMET30*Δ*WD40* transformants were screened via PCR with the primers *G418*-F and *G418*-R. Table S1. Formulations of the different culture media. Table S2. CgMET30cds sequence. Table S3. List of primers for RT–qPCR. Table S4. RT–qPCR amplification programme. Table S5. RT–qPCR reaction system.
