# Supplementary material for: Molecular Insights into the Role of the MET30 Protein and Its WD40 Domain in Colletotrichum gloeosporioides Growth and Virulence
Source: J Fungi (Basel). 2025 Jan 21;11(2):84. doi: 10.3390/jof11020084 (PMC11855936; doi:10.3390/jof11020084)
Supplement: Supplementary file 1 [file jof-11-00084-s001.zip › jof-3409487-supplementary.pdf]

Supplementary Materials

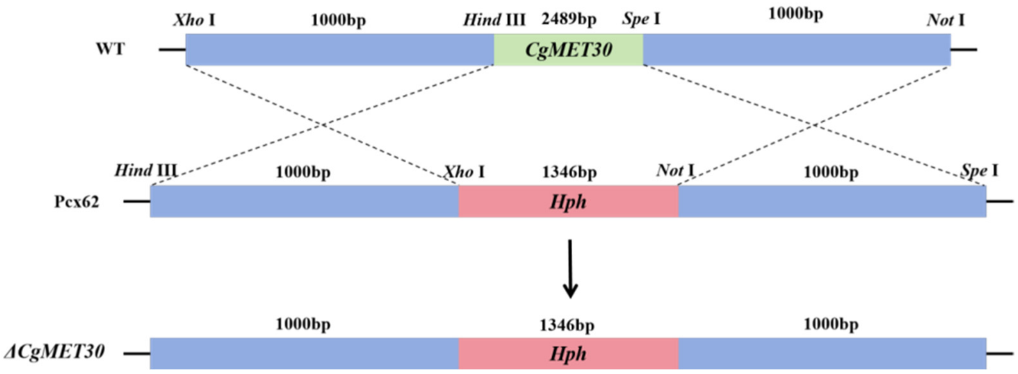

Figure S1. Diagram of homologous recombination structure.

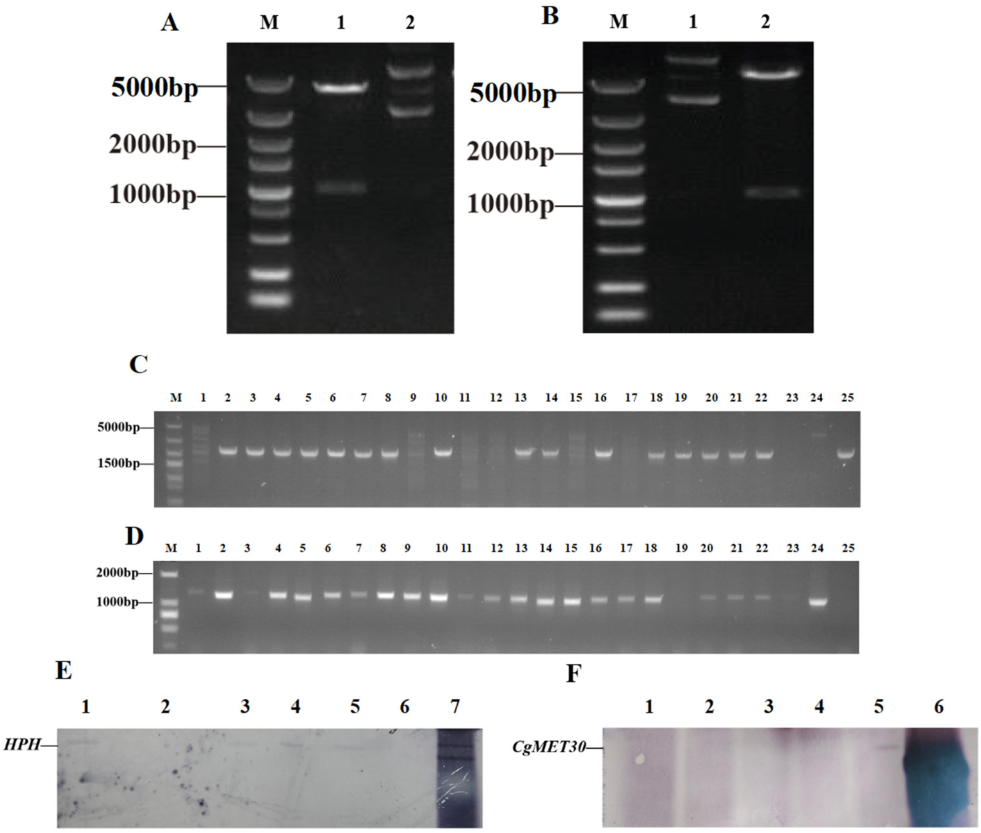

Figure S2. Gene knockout and validation. (**A**) The upstream arm of the knockout plasmid was identified by double enzyme digestion, where M is a 5000 bp marker, 1 is the plasmid pCX62::*CgMET30*-Up digested by Xho I and Hind III, and 2 is the pCX62::*CgMET30*-Up plasmid. (**B**) The downstream arm of the knockout plasmid was identified by double enzyme digestion, where M is a 5000 bp marker, 1 is the pCX62::*CgMET30*-Down plasmid, and 2 is the plasmid pCX62::*CgMET30*-Down digested by Spe I and Not I. (**C**) Transformant screening via PCR using the *CgMET30*-F1/*CgMET30*-R primer pairs, where M is a 5000 bp marker, 1~23 are *CgMET30* partial knockout transformants, 24 is the pCX62::*CgMET30* plasmid, and 25 is WT CSLL-11 genomic DNA. (**D**) Transformant screening via PCR using the *HPH*-F/*HPH*-R primer pairs, where M is a 2000 bp marker, 1~23 are *CgMET30* partial knockout transformants, 24 is the pCX62::CgMET30 plasmid, and 25 is WT CSLL-11 genomic DNA. (**E**) Hph probe hybridization, where 1~5 are transformants Δ*Cgmet30*-2, Δ*Cgmet30*-10, Δ*Cgmet30*-13, Δ*Cgmet30*-17, and Δ*Cgmet30*-19, respectively, 6 is the WT, and 7 is the knockout vector. (**F**) *CgMET30* probe hybridization, where 1~4 are transformants Δ*Cgmet30*-2, Δ*Cgmet30*-13, Δ*Cgmet30*-17, and Δ*Cgmet30*-19, respectively, 4 is the WT, and 5 is the *CgMET30* PCR product.

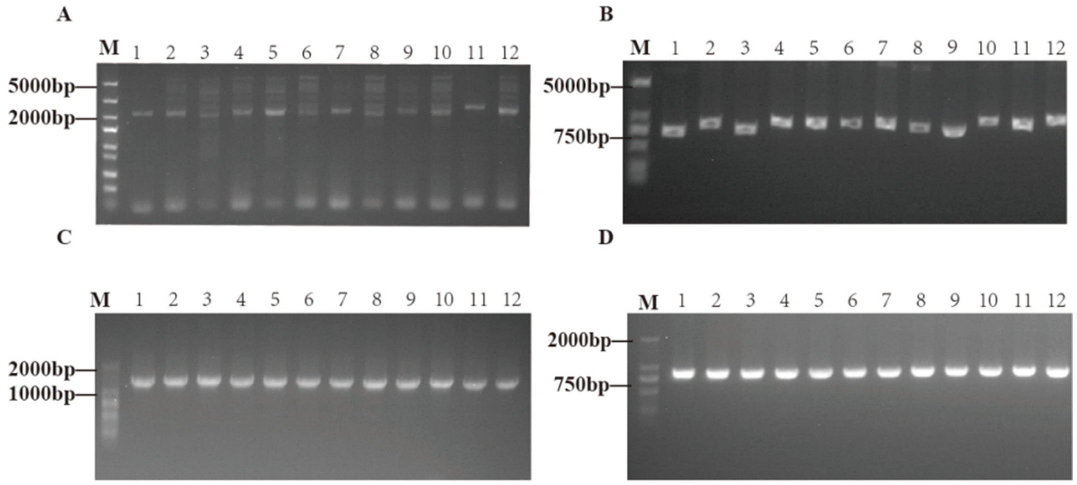

Figure S3. (**A**) Complementary *CgMET30* transformants were screened via PCR with the primers *CgMET30*-1F and *CgMET30*-R. (**B**) Complementary *CgMET30* transformants were screened via PCR with the primers *G418*-F and *G418*-R primers. (**C**) Complementary *CgMET30ΔWD40* transformants were screened via PCR with the primers *CgMET30*-1F and *CgMET30*-R. (**D**) Complementary *CgMET30ΔWD40* transformants were screened via PCR with the primers *G418*-F and *G418*-R.

Table S1. Formulations of the different culture media

Medium Name	Formulation	Sterilisation Condition
PDA	46 g/L, Antibiotic-free dextrose potato medium prepared by Solarbio was purchased.	121°C, 20 min
CM	50 mL 20×Nitrate Salts, 1 mL Trace Elements, 10 g/L D-Glucose, 2 g/L Peptone, 1 g/L Yeast Extract, 1 g/L Casamino Acids, 1 mL Vitamin Solution, 15% Agar; 20×Nitrate Salts: 120 g/L $NaNO_3$, 10.4 g/L KCl, 10.4 g/L $MgSO_4{\cdot}7H2O$, 30.4 g/L KH_2PO_4, Store at 4 °C; 1000×Trace Elements: 2.2 g/L $ZnSO_4{\cdot}7H2O$, 1.1 g/L H_3BO_3, 0.5 g/L $MnCl_2{\cdot}4\ H_2O$, 0.5 g/L $FeSO_4{\cdot}7H_2O$, 0.17 g/L $CoCl_2{\cdot}6H_2O$, 0.16 g/L $CuSO_4{\cdot}5H_2O$, 0.15 g/L $Na_2MnO_4{\cdot}2H_2O$, 5 g/L Na_4EDTA, Store at 4°C; Vitamin Solution: 0.01 g/L Biotin, 0.01 g/L Pyridoxin, 0.01 g/L Thiamin, 0.01 g/L Riboflavin, 0.01 g/L PABA (Para-aminobenzoic acid), 0.01 g/L Nicotinic Acid, Store at 4 °C in the dark.	121°C, 15 min, NaOH to pH to 6.5
OM	50 g/L oatmeal, dissolved with dd H_2O, filtered through a layer of gauze, added 15% agar	121°C, 20 min
V8	100 ml V8, 0.2 g/L $CaCO_3$, 15% agar	121°C, 20 min
SDC	100 g/L of rice straw, boiled for 20 min, filtered through two layers of gauze, discarded the residue, added 40 g/L of corn flour and 15% agar	121°C, 20 min

Table S2. CgMET30cds sequence.

ATGCCCTCGTCGACCACTCCTTTCGACGACGACGAAGAAGACGCAGACGACGACG<br>ACGAGATGGCTCCACGCATCTACAACGTTTCCGACTGGGCCCCGACTCAGGAGGCC<br>ATCTACGACGAGCCGTCCTCCAATTCCGCCATCCAAGAAGACGAAGCCGGCGGAC<br>AGCAGCTTCAAACTACTACTAGACCCTCCTTTCAACAGCACCGCCTTCGCAGCATG<br>AGCCTCTCTCTCCCATGGCGGCGCAAGTCGTTCATGCTCCACGCCGATACCGACCTG<br>GCCAGCCATATGGAAGACCTTACTCTCGAGCGATCACGAAGCCACGGCGGCCCCA<br>GCAACGAGCAGAACCCGGCCCAGGGTACCAACGCGACAGCACAAGGCAATGGAA<br>GCATCAAAGGAATGATCCGTCGGGCATCAGTCTCGCTAAAGGGCATCGTCCATCAC<br>AAGCGACACTCCATCGGCGGTGCTCACCACGAGATCCCGAGTCAGCATCACGGCC<br>ATCGCCCGACCACTTCACACTCCCACTTCCCCTGGCAACGCCTTCGTCAGGCTGCCA<br>GCTTCCGCCACTCCAAGTCATCCCATGGCTTCGACGCCAACAGCGAGGCATCCTCTT<br>CTGGCCAGTGTTTCCCATGTATGCCCCTTCCCGGCATCGGCGGAGAGCCTCCCGTCA<br>TTCCGTACCACACCGGTGCCGCGGCGAAGGCTGCCGCCGCGGCGCAGAACCAGAT<br>GTTGCAGAACAAGTGGCTTCGTGCTTCCCAGGACGATTTCACCGACCGCGAGAGCG<br>GTATCGGCATCACTGTTACCAGCGCCGAATCAGAGATCGAGCTGGAGGCCGAGGA<br>CGCCAACATCAGCAGGACAGACTTCATCTCCCAGCTCCCAGTGGAGCTCGCCATCC<br>AAGTACTCGCCCATTTGGACGCCACGGCGCTCAACACCGCAAGTAGAGTTTCCCGC<br>TACTGGCAGGGAATGGTACAAAACCAGCACGTCTGGCGCGAGTCCTTCCTGCGAGA<br>AAAGACGGGCACCTATGCGACGAGTCTTCCCGTTCAGCCGGGGACCGGCCAGGGT<br>GTGCCCGCCATCACGCCCGGTGTCGATTGGAAGCAGATCTACAAGTCCAAGCAGG<br>AGCTCGACTTGCGATGGAAAGAAGGCAAGGCGAGACCAGTGTATCTCAACGGGCA<br>CTTGGATAGTATCTACTGCCTGCAGTTTGATGAATCGAAAATCATCACTGGCTCTCG<br>TGACAAGACCATTCGCATTTGGGACATGCGGACGTTCGAATGCCGACTTGTCATCG<br>GTCCTCCTGAAGTTGTCAACGACATCTCCTTGCTGATTGATGAAGATGGCTTGCCGA<br>TTCACTACGCAACCCTGCCTGACAACCCGCGCGCGAAACCGTCGACGCCTGCTCTG<br>GTTTCCTTCCCGACTCACCACAAAGCATCCATCCTCTGCCTGCAGTATGATGAGCGC<br>ATCCTGGTGACGGGCTCCTCCGACTCGACTTGCATCGTGTACAACGTCCGCTCTGGA<br>TACCGGCCGATTCGTCGTCTCCGTCACCACACTGCGGCCGTCCTTGACCTCGCATTC<br>GACGACAAGCACATCATCACCTGCAGCAAGGACATCAGCATCTGCGTCTGGGACC<br>GTGCTACAGGGCAGCTTCTTCGTCAACTTCGTGGGCACTCAGGCCCTGTCAACGCC<br>GTTCAGATGCGTGGAAACACCATCGTCTCGTGCTCCGGCGACTTCCGTGTAAAGCTT<br>TGGAACATTGACACCGGCAAGAACATCCGCGAATTCCAGGGCCACACCAAGGGTT<br>TGGCGTGCTCTCAATTCTCTGAGGATGGCCGCTACGTTGCGTCAGCCGGCAACGAC<br>AAGGTCATCCGGATTTGGGACGCCAACACGGGCGAGTGTCTCCGCGAAATGAAGG<br>CCCACGACAACCTTGTGCGCAGCCTGCACATCGACTCTGTAAGCGGTCGTCTTGTCA<br>GCGGCAGCTACGACACCGACATCAAGGTTTTCGATATGGAGACCGGTCGGCAGCTG<br>CTCGACTTCCCGCGTTGGCATGCTAGCTGGGTGCTGAGCGCGAAGAGCGACTACCG<br>TCGGATTGTCAGCACCGGTCAGGACCCCAAGATCCTGATCATGGACTTCGGCGCCG<br>ATGTTCCGGGCATCGAGAATCTCGAGAGCGGCGGCGCTTGCATGATCGACGGCGG<br>ATACATCTAG

Table S3. List of primers for RT-qPCR

Primer name	Sequences (5′~3′)	Tm(℃)
Actin-RT-F	GATCACTTCTCTTGCTCCTTCTTCC	62
Actin-RT-R	TCTCGTCGTACTCCTGCTTGG	
ncr2-RT-F	ATGCCTGCCAGCCGAACCCATC	60
ncr2-RT-R	GAAGAAGCTGCTTCCGAATT	
Abr2-RT-F	TATATCGGTGGATCGCTGGA	60
Abr2-RT-R	TTAGTGGAGGAGAGCACTCG	
CWI5-RT-F	CCTTGACCACCAGCACTGAAAC	62
CWI5-RT-R	CAGGAGAAGCGGCACCAGAG	
CWI8-RT-R	CCGCTGCTGTTGAGGTTGAG	62
CWI8-RT-R	GACCGTAGAGGATGCCAATGC	

Table S4. RT－qPCR amplification program

Temperature（℃）	Time	Number of cycles
95	5 min	1
95	10 s	
60	40 s	40×
95	15 s	
60	1 min	1
95	30 s	1

Table S5. RT－qPCR reaction system

Reactant	Volumes（μL）
Forward primer（0.25 μM）	0.4
Reverse primer（0.25 μM）	0.4
ChamQ Universal SYBR qPCR Master Mix	10
cDNA	1
ddH2O	8.2
